# The development and validation of the Questionnaire on Anticipated Discrimination (QUAD)

**DOI:** 10.1186/1471-244X-13-297

**Published:** 2013-11-07

**Authors:** Jheanell Gabbidon, Elaine Brohan, Sarah Clement, R Claire Henderson, Graham Thornicroft

**Affiliations:** 1 Health Service and Population Research Department, King’s College London, Institute of Psychiatry, De Crespigny Park, London SE5 8AF, UK; 2 Adelphi Values, Adelphi Mill, Bollington, Cheshire SK10 5JB, UK

**Keywords:** Measure, Anticipate, Discrimination, Stigma, Psychometric

## Abstract

**Background:**

The anticipation of mental health-related discrimination is common amongst people with mental health problems and can have serious adverse effects. This study aimed to develop and validate a measure assessing the extent to which people with mental health problems anticipate that they will personally experience discrimination across a range of contexts.

**Methods:**

The items and format for the Questionnaire on Anticipated Discrimination (QUAD) were developed from previous versions of the Discrimination and Stigma Scale (DISC), focus groups and cognitive debriefing interviews which were used to further refine the content and format. The resulting provisional version of the QUAD was completed by 117 service users in an online survey and reliability, validity, precision and acceptability were assessed. A final version of the scale was agreed and analyses re-run using the online survey data and data from an independent sample to report the psychometric properties of the finalised scale.

**Results:**

The provisional version of the QUAD had 17 items, good internal consistency (alpha = 0.86) and adequate convergent validity as supported by the significant positive correlations with the Stigma Scale (SS) (r = 0.40, p < 0.001) and the Internalised Stigma of Mental Illness Scale (ISMI) (r = 0.40, p < 0.001). Three items were removed due to low endorsements, high inter-correlation or conceptual concerns. The finalised 14 item QUAD had good internal consistency (alpha = 0.86), good test re-test reliability (ρ_c_ = 0.81) and adequate convergent validity: correlations with the ISMI (r = 0.45, p < 0.001) and with the SS (r = 0.39, p < 0.001). Reading ease scores indicated good acceptability for general adult populations. Cross-replication in an independent sample further indicated good internal consistency (alpha = 0.88), adequate convergent validity and revealed two factors summarised by institutions/services and interpersonal/professional relationships.

**Conclusions:**

The QUAD expanded upon previous versions of the DISC. It is a reliable, valid and acceptable measure which can be used to identify key life areas in which people may personally anticipate discrimination, and an overall tendency to anticipate discrimination. It may also be useful in planning interventions aimed at reducing the stigma of mental illness.

## Background

A classic definition of stigma provided by Goffman is “an attribute that is deeply discrediting”. It is further noted that this attribute reduces the individual “from a whole and usual person to a tainted, discredited one” p.3. [1]. This suggests a socially constructed nature of stigma in which individuals may become devalued in a particular social context. Goffman further noted that this fear of becoming “discredited” can have major impacts on people’s lives leading them to conceal the stigmatised condition or avoid situations in which they might be stigmatised [2]. In this case, the affected group are people with mental health problems.

There are different aspects of stigma such as perceived stigma, self stigma and experienced stigma and there are many levels at which stigma may operate [3,4]. However, the concept of anticipated discrimination does not appear to be adequately covered by existing definitions of stigma. This concept refers back to Goffman’s early notion of the fear of becoming personally 'discredited’ based on a stigmatising condition and the associated negative consequences [1], but draws upon the more contemporary concept of discrimination (unfair treatment). The perceptions that people with mental health problems have that they will be viewed negatively or treated unfairly may result in avoidant behaviour in situations where stigma and discrimination is anticipated [2,5]. Furthermore, the expectation of such rejection refers to the anticipated responses of others which may or may not be as a result of an actual experience of discrimination [6,7].

Research suggests that anticipated stigma may be a common experience for people with mental health problems. In a German survey examining both anticipated and experienced stigma, people with a diagnosis of schizophrenia or depression reported high levels of anticipated stigmatisation in accessing employment roles. These were 69% and 81.5% respectively [8]. However, only a small proportion of these individuals had experienced such stigmatisation, 19% and 1.9% respectively.

In relation to the anticipation of unfair treatment, an international study found that over a third of participants reported anticipated discrimination when seeking employment and close personal relationships having no experience of such discrimination [6]. The same study investigated the level of anticipated discrimination in people with schizophrenia across 27 different countries and employed the use of four items from the Discrimination and Stigma Scale (DISC-10) to assess anticipated discrimination. Due to anticipated discrimination, participants reported that they had stopped themselves from applying for employment, training or education (64%) and looking for close relationships (58%). Feeling the need to conceal their diagnosis (72%) was also highly related to their expectation of avoidance by others if they knew about their diagnosis [9]. Furthermore, 47% of people with major depressive disorder who anticipated discrimination in finding or keeping a job and 45% in their intimate relationships had not experienced discrimination [10]. The findings of these studies demonstrate that anticipated discrimination is not only common amongst people with schizophrenia or depression but more importantly that this is not necessarily related to experienced discrimination.

These results emphasise the importance of differentiating between anticipated and experienced stigmatisation as separate constructs and the relevance of this process in planning stigma reducing interventions [8]. It is not only important to introduce measures which reduce the discrimination experienced from others but also the discrimination anticipated by the individuals themselves [9]. It can be expected that by addressing anticipated stigmatisation or discrimination as part of a public health intervention to reduce stigma, the anticipation of discrimination and its negative consequences may decrease following or at the same time as a reduction in public stigma [4]. Consequently, greater understanding of the contexts in which people anticipate stigma and discrimination is needed. However, there are no current scales which focus specifically on measuring personally anticipated stigma or discrimination of people with mental health problems across a wide range of contexts.

The anticipated discrimination when seeing a psychiatrist scale (ADSP) produced three distinct factors relating to 'anticipated discrimination’, 'anticipated job problems’ and 'anticipated shame’ [11]. However, this scale was specifically developed to assess anticipated discrimination associated with help seeking for a mental health problem. There are also several scales which include items similar to anticipated discrimination including the perceived discrimination and devaluation scale (PDD), the stigma scale (SS) and the internalised stigma of mental illness scale. Items of the PDD scale such as *“Most young women would be reluctant to date a man who has been hospitalized for serious mental disorder”*[12], refer to the anticipation of most other people and lacks personal relevance [13]. Furthermore, perceived discrimination scales ask respondents to report the extent to which they perceive 'people with mental illness’ would be discriminated against rather than their perceptions of how likely it is that they themselves will be discriminated against. Three disclosure items of the SS refer to anticipated discrimination in personally relevant life areas (employers, friends and neighbourhood). However, the remaining seven disclosure items of the SS refer to the management of disclosure to avoid discrimination in general contexts with items such as *“I am scared of how other people will react if they find out about my mental health problems”* and *“I avoid telling people about my mental health problems” *[14]. The social withdrawal subscale of the ISMI refers to efforts to avoid social situations in order to minimise burden to others *(“I stay away from social situations in order to protect my family or friends from embarrassment”)* or to avoid detection of symptoms. Other items refer to avoidance in more general contexts such as *“I avoid getting close to people who don’t have mental illness to avoid rejection”*[15]. Whilst the aforementioned scales contain items referring to anticipated discrimination, they are limited to a small number of areas of life in which people with mental health problems may personally anticipate stigmatisation or discrimination.

The subsection of the DISC-10 employed in the International Study of Discrimination and Stigma Outcomes (INDIGO) assesses some aspects of anticipated discrimination, including avoidant behavioural responses in the context of work and close personal relationships as well as a disclosure item [6]. However, again these items are restricted to a small number of contexts and focus on responses to anticipated discrimination rather than the anticipation itself. This study drew upon the existing items of the experienced discrimination subscale of the DISC-10 to develop a measure of anticipated discrimination (Questionnaire on Anticipated Discrimination-QUAD) which covers a broad range of contexts in which stigma may be personally anticipated.

### Aims

To develop a scale to assess the extent to which people with mental health problems anticipate personally experiencing mental health-related discrimination across various domains of life. A further aim of this study is to establish the psychometric properties of this scale: the Questionnaire on Anticipated Discrimination (QUAD).

## Method

### Design

The design is a four-stage psychometric validation study. Stage one involved the development of QUAD where initial items were formulated based on previous versions of the DISC followed by two focus groups to formulate any further items and refine the measure. There were also cognitive debriefing interviews to finalise the wording and format of the provisional version of the QUAD. Stage two was a cross-sectional online survey in which respondents completed the provisional QUAD and other measures. In this stage a subsample re-completed the provisional QUAD online two weeks later to assess test-retest reliability. In stage three the research group considered the findings, excluded some items and re-ran the analyses in order to finalise the QUAD. Finally, in stage four the analyses were re-run to establish the psychometric properties of the finalised QUAD in an independent sample and to explore its factor structure.

### Sample and recruitment

The inclusion criteria for participants in stages one to three of the study were: 1) in contact with secondary mental health outpatient services currently or in past 12 months; and 2) aged 18 years or older. The eligibility criteria for participants in stage four of the study were: 1) aged 18 years or older; 2) a clinical diagnosis of Depression, Bipolar disorder or Schizophrenia spectrum disorders; 3) self-defined Black, White or Mixed (either Black or White) ethnicity; and 4) current treatment with a community mental health team.

Participants in the focus groups and cognitive debriefing interviews (stage one) were mental health service users living in South East England invited through the Mental Health Research Network (MHRN). Participants for the main study (stages two and three) were recruited through advertisements on the websites of two national mental health charities (Rethink Mental Illness, Mental Health Foundation) and an advertisement on a social network page (Facebook) of a national anti-stigma campaign (Time to Change). The target sample size for the online survey was 100, which was the required number to perform the analyses outlined as part of the psychometric validation of QUAD. A sample size of 40 was sufficient to establish that the test-retest reliability is at least 0.7, assuming that the true level is 0.8 (where α = 0.05; β = 0.20; number of replications = 2) [16]. Participants in stage four were from the MIRIAD (Mental Illness-Related Investigations on Discrimination) study (part of the SAPPHIRE Research Programme on Stigma and Discrimination in Mental Health - http://www.sapphire.iop.kcl.ac.uk[17]). This was a cross-sectional study of 202 individuals using secondary mental health services in South London. Following approval from members of the community mental health teams (n = 14), the caseloads were screened based on the eligibility criteria and eligible service users were sent a letter inviting them to contact the research team if they were interested in taking part.

### Procedure

The QUAD scale was developed using an iterative process. In stage one an early version of QUAD was developed following an examination of previous versions of the DISC scale [6,18] and on further work by our group examining areas of life where discrimination may be personally experienced not covered in the DISC [19,20]. Two focus groups took place in order to obtain feedback on the initial version of the QUAD and cognitive debriefing interviews were conducted in order to understand the mental process used when answering the questions and to further refine item wording and format, if necessary. In the focus groups participants were shown a PowerPoint presentation about the intial version of the QUAD, asked to give their overall opinions and to discuss the concept of anticipated discrimination. They were also asked to consider the format including the draft response options and whether they found any question particularly upsetting or distressing. Cognitive debriefing interviews involved the researcher going through the initial QUAD with each individual service user and obtaining feedback about the measure [21]. In each component of stage one, participants were asked to provide socio-demographic information including details about age, gender and ethnicity as well as self-reported clinical data.

In stage two written informed consent was obtained from the participants in the main study prior to the start of the survey. They were required to complete the provisional version of the QUAD along with other measures included in the online survey. Items on participants’ socio-demographic and clinical characteristics were also included. Those who volunteered to complete the QUAD for a second time were sent a link two weeks later to complete the measure in order to obtain test-retest data. Each participant received a £10 voucher in return for their time. The aim of the online survey was primarily to further develop and validate the QUAD but was also used to validate an additional unrelated scale in development (Barriers to Access to Care Evaluation - BACE) [22]. The online survey data were analysed to assess the psychometric properties of the provisional QUAD.

In the third stage of the development the research group considered the psychometric and conceptual properties of the provisional QUAD, excluded a small number of items on the basis of these findings, and re-ran the analyses in order to produce a finalised version of the QUAD.

In stage four the psychometric properties of the finalised QUAD were re-established using data from the MIRIAD study. Written informed consent was obtained and participants were interviewed over two sessions (range 1–4) by Research Assistants. The MIRIAD interview schedule collected demographic and clinical information and contained a battery of measures on stigma and discrimination including the finalised 14 item QUAD, DISC and ISMI. Participants in this stage received £15 as a thank you for their time. As a final step we carried out an exploratory factor analysis using the online survey sample (stage two) to undertake a preliminary replication of the factor structure found in the larger independent sample.

### Measures

The version of the *Questionnaire on Anticipated Discrimination Scale (QUAD)* used in the online survey is a self-completion measure containing 17 items addressing areas of anticipated discrimination. Participants are asked to rate whether they expect to be treated unfairly in various areas of life if people know about their mental health problem. Each item requires a response on a four-point Likert scale ranging from 0 (strongly disagree) to 3 (strongly agree) and includes items such as “If friends know about my mental health problem they will treat me unfairly”. A mean score is calculated by adding each item score and dividing by the number of applicable, non-missing items in addition to a count score of the number of areas of life in which individuals expect anticipated discrimination.

The *Stigma Scale* (SS) is a 28-item measure used to assess the stigma of mental illness. The scale comprises of three sub scores which include discrimination (12 items), disclosure (11 items) and positive aspects of mental illness (5 items) [14]. Each item requires a response on a five-point Likert scale ranging from “strongly agree” to “strongly disagree”. The scale has good internal consistency (α = 0.87) and adequate test-retest reliability (kappa range 0.49 – 0.71) [14].

The *Internalised Stigma of Mental Illness Scale (ISMI)* is a 29-item measure that assesses experiences of internalised stigma in mental health service users. The scale is composed of five subscales which include alienation, stereotype endorsement, perceived discrimination, social withdrawal and stigma resistance. Items require a response on a four-point Likert scale ranging from “strongly agree” to “strongly disagree”. Strong internal consistency (α = 0.87) and test-retest reliability (r = 0.92) have been reported for the scale [15].

The *Discrimination and Stigma Scale (DISC)* is measure of discrimination experienced based on having a mental health problem. The DISC has a 4-point Likert scale (not at all; a little; moderately; a lot) and assesses experiences of discrimination across 21 life domains including family, friends, dating or intimate relationships, housing and employment. The scale has been found to have good reliability, validity and acceptability [18].

Participants in the online study were also asked to rate the QUAD on a scale from 1 (very poor) to 10 (very good) and were given the opportunity to comment on the overall scale and individual items.

### Analysis

Data were analysed using SPSS v15 and STATA v10. The psychometric properties of the provisional and final versions of the QUAD were established by assessing the reliability, validity, precision and acceptability of the measure.

#### *Reliability*

The internal consistency of the QUAD was assessed using Cronbach’s alpha with a criterion of 0.70 taken as indicating good internal consistency [23]. In order to assess test-retest reliability of individual items, weighted kappa coefficients were calculated with values above 0.40 indicating moderate agreement [24,25]. Lin’s concordance was used to calculate the overall test-retest reliability for the QUAD between first administration and follow up. A criterion of Lin’s ρ_c_ ≥0.70 was used to indicate acceptable reliability [26].

#### *Validity*

Construct validity was established by assessing convergent validity where it was anticipated that there would be significant moderate positive correlations between the QUAD, the Stigma Scale, the ISMI and the DISC. The criterion for acceptable convergent validity was indicated by correlation coefficient values between 0.40 and 0.70 [27]. In stage four construct validity was further assessed by conducting an exploratory factor analysis. A subject to item ratio of 10:1 was taken as an adequate sample size for conducting factor analysis [28,29]. The 14 items were analysed based on principal axis factoring with oblique (promax) rotations as it was considered that the factors would be correlated. The problems with retaining factors with eigen values greater than one have been noted and there is consensus in the literature that this is among the least accurate methods [30-32]. The scree test has been recommended as a better alternative, therefore the scree plot was examined to identify the natural break point in the data as an indicator of the number of factors to be retained [31]. Items with factor loadings > 0.3 was taken as the criteria for acceptable validity [33].

#### *Precision*

This refers to how well each item fits within the proposed scale and the appropriateness of the scaling assumptions. Corrected item-total correlations were examined where a correlation < 0.30 was indicative of unacceptable fit of the items with the total score of the scale [34].

#### *Acceptability*

In order to establish the extent to which the scale was acceptable for its intended population the following forms of acceptability were examined: 1) maximum endorsement frequencies (MEF), 2) aggregate adjacent endorsement frequencies (AEF), 3) Flesch reading ease score and 4) respondent opinion. In considering MEF, the N (%) of respondents endorsing each response category was established. MEF >80% in any category indicates that the item may need further consideration [35]. In addition, a violation of the MEF criterion at either the top or bottom end of the scale may indicate ceiling and floor effects respectively. AEF assesses the proportion of responses in two or more adjacent scale points of an item, where the criterion of >10% was considered acceptable [35].

The Flesch reading ease score and the Flesch-Kincaid Grade level functions in Microsoft Word were calculated in order to establish whether the wording of the scale was suitable for the target audience. Reading ease scores range from 0–100 with scores of 60–70 indicating acceptability for general adult populations. Grade level scores indicated the US educational grade to which the document is most appropriate [36]. In order to assess the level of participant burden, participants in the online study were asked to rate their level of satisfaction with the QUAD on a scale from 1 (very poor) to 10 (very good) and given the option to provide further opinions on the scale.

### Ethics

The QUAD study protocol was approved by the King’s College London Psychiatry, Nursing and Midwifery Research Ethics Sub-Committee (ref: PNM/09/10-103). The MIRIAD study was approved by the East of England/Essex 2 Research Ethics Committee (ref 11/EE/0052).

## Results

### Participants

Twenty five individuals participated in stage one: twenty people took part in the service user focus groups and five participated in the cognitive debriefing interviews. The mean age of participants was 54 years old (sd = 12.69), with an age range of 32 to 75 years. There were 13 female (52%) and 11 male (44%) participants and the majority (76%; n = 19) reported their ethnicity as White British, Irish or any other White background. Four percent (n = 1) reported their ethnicity as Black Caribbean, 12% (n = 3) Indian or any other Asian background and 8% (n = 2) as mixed ethnicity. Bipolar disorder (24%; n = 6) and schizophrenia/schizo-affective disorders (20%; n = 5) were the most commonly self–reported diagnoses.

One hundred and seventeen individuals completed the online survey in stage two, and 59 of these participants completed the QUAD at the second time point to provide test-retest reliability data. There was no significant difference between the main sample and those providing test-retest data in relation to age, gender, ethnicity, education, employment status, age at first treatment, and hospital admittance for psychiatric treatment. However, those with non-psychotic conditions were more likely to be in the retest sample (66% vs. 34%, Χ^2^ = 5.06, p = 0.02) compared to those with psychotic conditions. The characteristics of the participants in stages one and two are shown in Table 1. The mean age of participants in stage two was 36 years, with a range of 18 to 70 years. Eighty percent were female, the majority reported their ethnicity as White British (87%), and 42% were in full or part-time employment. Depression (34%) and bipolar disorder (31%) were the most common self-reported primary diagnoses and 46% had been hospitalised for a mental health problem.

**Table 1:** Participants’ sociodemographic and clinical characteristics

**Stage 1 participants**			
**Variable**		**N**	**%**
Gender (n = 24)	Male	11	44.0
	Female	13	52.0
Ethnicity (n = 25)	White British	15	60.0
	White Irish	2	8.0
	Other white background	2	8.0
	Black British/Black African	1	4.0
	Indian/Bangladeshi	2	8.0
	Other Asian background	1	4.0
	Mixed	2	8.0
Age (n = 25)	Mean (sd) = 54.24 (12.69)	Range = 32 - 75	
Self-reported diagnosis (if more than one, first listed) (n = 20)	Schizophrenia/schizoaffective disorder	5	20.0
	Bipolar disorder	6	24.0
	Personality disorder	3	12.0
	Depression	3	12.0
	Obsessive compulsive disorder	2	8.0
	Unipolar disorder	1	4.0
**Stage 2 participants**			
**Variable**		**N**	**%**
Gender (n = 117)	Male	24	20.5
	Female	93	79.5
Ethnicity (n = 117)	White British	102	87.2
	White Irish	5	4.3
	Other white background	5	4.3
	Black British/Black African	2	1.8
	Indian/Bangladeshi	2	1.8
	White and Asian	1	0.9
Age (n = 115)	Mean (sd) = 36.1(11.1)	Range = 18 - 70	
Highest level of education (n = 117)	Higher education qualification/degree	61	54.7
	Vocational qualification	16	13.7
	A levels	17	14.5
	GCSE/O level/CSE	19	16.2
	No formal qualifications	1	0.9
Employment status (n = 117)	Work full-time	33	28.2
	Work part-time	16	13.7
	Volunteer	19	16.2
	Looking after own children	2	1.7
	Student	11	9.4
	Retired	1	0.9
	Not working	35	30.0
Relationship status (n = 116)	Single	52	44.8
	Married/civil partnership/cohabiting	46	39.7
	Divorced or separated	16	13.8
	Widowed	2	1.7
Any children (including adult and non-resident children) (n = 116)	Yes	34	29.3
	No	82	70.7
Self-reported diagnosis (if more than one, first listed) (n = 107)	Schizophrenia/schizoaffective disorder	5	4.7
	Bipolar disorder	33	30.8
	Depression	36	33.6
	Anxiety disorder	13	12.1
	Personality disorder	17	15.9
	Other	3	2.8
Ever admitted to hospital for psychiatric treatment? (n = 116)	Yes	53	45.7
	No	63	54.3
Any involuntary hospital admissions? (n = 114)	Yes	12	10.5
	No	102	87.2
Years since first treatment for mental health problem (n = 105)	Mean(sd) = 23.03 (9.8)	Range = 4 - 52	

Two hundred and two individuals took part in the MIRIAD study (stage four). The demographic and clinical characteristics of the participants in stage four are shown in Table 2. The mean age of participants in stage four was 41 years (range = 19–67), 53.5% self-defined their ethnicity as White, 38.1% as Black and 8.4% as Mixed. The majority (47.5%) had a primary diagnosis of Schizophrenia spectrum disorders, 32.2% had a diagnosis of Depression and 20.3% had a diagnosis of Bipolar disorder.

**Table 2:** MIRIAD study participants’ socio-demographic and clinical characteristics

**Variable**	**Categories**	**Total n = 202**	**Percentage**
Gender	Male	92	45.5
	Female	110	54.5
Ethnicity expanded categories (self-defined)	White British	92	45.5
	White other	16	7.9
	Mixed	17	8.4
	Black British	24	11.9
	Black African	28	13.9
	Black Caribbean	25	12.4
Age (years)	Mean (sd; range)	202	41.8 (11.1; 19–67)
Employment status	Employed	46	22.8
	Not employed	126	62.4
	Student/Training/Volunteer	25	12.4
	Missing	5	2.5
Education level	No qualifications	25	12.4
	Qualifications usually taken at age 16	50	24.8
	A-levels/Vocational	67	33.2
	Degree or higher	60	29.7
Relationship status	Single	128	63.4
	Married/Partner	45	22.3
	Divorced/Widowed	29	14.4
Clinical diagnosis from case records	Bipolar disorder	41	20.3
	Depression	65	32.2
	Schizophrenia	96	47.5
Psychiatric hospital admissions	Ever admitted to hospital	139	68.8
	Admitted in last 12 months	35	17.8
	Compulsory admission in last 12 months	14	6.9
			Mean (sd; range)
Years since first contact with mental health services		201	15.1 (11.1; 0–46)

### Stage one: provisional version of the QUAD

Following the development and psychometric validation of the DISC scale, there were recommendations to develop a measure of anticipated discrimination [18]. The initial items of QUAD were based on the domains in the DISC which measures personally experienced discrimination. Items in the DISC such as 'Have you been treated unfairly in making or keeping friends?’ were adapted to focus on the anticipation rather than the experience of discrimination ('If friends know about my mental health problem they will treat me unfairly’). A total number of 17 personal life domains (friends, neighbours, dating/intimate relationships, housing officials, education, spouse/partner, family, employers, work colleagues, transport drivers and officials, religious community, police, physical health staff, mental health staff [18], benefit officials [6], parents of other children [19] and children and teenagers in the street [20]) were adapted and expanded from versions of the DISC scale and further work by our research group to form the initial version of QUAD.

The overall opinion of the participants in the focus group was that the wording and format was good and the domains were considered relevant. The media was suggested as an important domain, however, this was not considered to be a personal life area and therefore not included. Participants also suggested the inclusion of a 'neither agree nor disagree’ category which was added to the version later used in the cognitive - debriefing interviews. Participants in the cognitive - debriefing interviews found that the instructions were clear, that the domains made sense hypothetically and were distinguishable. Furthermore, the 'neither agree nor disagree’ category appeared to be overused during the cognitive-debriefing interviews so was removed following this stage. This was to avoid overuse of this category which was a risk given that the anticipation of discrimination refers to hypothetical situations.

### Stage two: psychometric properties of the provisional version of the QUAD

#### *Reliability*

The Cronbach’s alpha value for the 17 item provisional QUAD scale was 0.86 indicating good internal consistency. All items were retained as when any of the items were deleted, the alpha value remained above 0.8. Eleven of the 17 items had kappa values between 0.61 and 0.80 indicating substantial agreement between test and retest; one item (item 8 - If employers know I have a mental health problem they will treat me unfairly) had a value above 0.80 indicating almost perfect agreement and the remaining five items had values between 0.41 to 0.60 indicating moderate agreement. Lin’s concordance for the provisional QUAD 17 items was ρ_c_ = 0.81 indicating substantial test-retest reliability [37].

#### *Validity*

The hypothesised significant moderate positive correlation between the QUAD and the Stigma Scale was supported (r = 0.40, p < 0.001). The QUAD was also significantly correlated with the ISMI scale (r = 0.40, p < 0.001), indicating a moderate positive correlation as hypothesised. Both correlations were within the required threshold of 0.40 to 0.70 indicating adequate convergent validity.

#### *Precision*

Corrected item-total correlations ranged from 0.26 to 0.66. One item (item 6 – 'If a spouse or partner knows about my mental health problem they will treat me unfairly’) fell outside the criterion with a correlation coefficient of < 0.30. However, inspection of Cronbach’s alpha if this item was deleted did not increase the overall reliability of the scale. The remaining 16 items satisfied the pre-specified criterion, indicating acceptable fit of these items with the total score of the scale.

#### *Acceptability*

Item 8 ('If employers know I have a mental health problem they will treat me unfairly’) was most highly endorsed for the “strongly agree” response category at 52.7% and item 16 ('If parents of children in my neighbourhood know I have a mental health problem they will treat me unfairly’) in the “agree” category at 53.9%. The MEF criterion was not violated as all response categories contained < 80% of responses. In addition, there was no violation of the MEF criterion at either the top or bottom end of the scale indicating no ceiling or floor effects. No items violated the AEF criterion of < 10% when considering the adjacent categories of “strongly agree” and “agree”, “agree” and “disagree”, “disagree” and “strongly disagree”. All adjacent categories contained > 10% of the responses on aggregate, indicating good acceptability.

The Flesch Reading Ease score for the QUAD was 70.3 which was just above the level of 60–70, indicating that it was slightly easier to read than documents intended for the general adult population. The Flesch-Kincaid Grade level was 7.3 indicating that it can be understood by 12–13 year olds. Respondents indicated positive opinions of the scale with a mean rating of 7.26 on the 10-point scale and participants generally commented that the scale was easy to complete.

### Anticipated discrimination

The percentage of respondents reporting anticipated discrimination for each of the 17 items is shown in Table 3. The most highly reported areas of life in which people anticipate discrimination were related to employers (item 8) and children and teenagers in my community (item 17), with 87.5% and 85.5% respectively endorsing both the “strongly agree” and “agree” response categories. These were followed by high levels of anticipated discrimination from parents of children in my neighbourhood (81.7%), work colleagues (81.7%) and people in my neighbourhood (78.4%). The least anticipated areas were “If mental health staff (e.g. psychiatrist, psychiatric nurse, social worker) know about my mental health problem they will treat me unfairly” (item 15), with 26.7% and “If a spouse or partner knows about my mental health problem they will treat me unfairly” (item 6), with 30.7%.

**Table 3:** Percentage of respondents reporting anticipated discrimination for each item in the provisional version of the QUAD

**Item no.**	**Statement**	**% (n)**
1.	If friends know about my mental health problem they will treat me unfairly	34.5 (40/116)
2.	If people in my neighbourhood know I have a mental health problem they will treat me unfairly	78.4 (91/116)
3.	If a person I want to date or have an intimate relationships with knows I have a mental health problem they will treat me unfairly	65.5 (74/113)
4.	If housing officials or landlords know I have a mental health problem they will treat me unfairly	73.9 (85/115)
5.	If teachers, lecturers or tutors know I have a mental health problem they will treat me unfairly	46.1 (53/115)
6.	If a spouse or partner knows about my mental health problem they will treat me unfairly	30.7 (35/114)
7.	If my family knows about my mental health problem they will treat me unfairly	49.6 (57/115)
8.	If employers know I have a mental health problem they will treat me unfairly	87.5 (98/112)
9.	If work colleagues know I have a mental health problem they will treat me unfairly	81.7 (94/115)
10.	If transport drivers and officials (e.g. bus driver, ticket inspector, taxi driver) know about my mental health problem they will treat me unfairly	56.5 (65/115)
11.	If benefit officials know I have a mental health problem they will treat me unfairly	60.9 (70/115)
12.	If religious officials or the community (e.g. at church, mosque or temple) know I have a mental health problem they will treat me unfairly	42.5 (48/113)
13.	If the police know I have a mental health problem they will treat me unfairly	63.8 (74/116)
14.	If physical health staff (e.g. GP, nurse, dentist) know I have a mental health problem they will treat me unfairly	43.2 (48/111)
15.	If mental health staff (e.g. psychiatrist, psychiatric nurse, social worker) know about my mental health problem they will treat me unfairly	26.7 (31/116)
16.	If parents of children in my neighbourhood know I have a mental health problem they will treat me unfairly	81.7 (94/115)
17.	If children and teenagers in my community know about my mental health problem they will treat me unfairly	85.5 (100/117)

% = strongly agree/agree response categories.

Note: Denominators less than n = 117 were due to missing data.

### Stage three: finalisation of the QUAD

Following the psychometric evaluation of the 17 item provisional QUAD, the research team considered these findings in order to finalise the QUAD. Item 6 (If a spouse or partner knows about my mental health problem they will treat me unfairly) fell outside the criterion of > 0.30 (r = 0.26). In addition, given the low endorsement of this item about discrimination from a partner/spouse (30.7%) and item 15 about discrimination from mental health staff (26.7%) and a conceptual concern that a spouse/partner and mental health staff would usually or always know about an individual’s mental health problem, these two items were deleted. The team also noted that there were three items relating to the neighbourhood: 1) people in my neighbourhood (item 2), 2) parents of children (item 16), and 3) children and teenagers (item 17). We considered that item 16 was a sub-set of item 2, further, on examining the inter-item correlation matrix found that this conceptual similarity was reflected in a high correlation coefficient (r = 0.57). Consequently item 16 was deleted.

### Psychometric properties of the final version of the QUAD

Following the removal of three items from the provisional version of the QUAD the analyses were re-run in order to establish the reliability, validity, precision and acceptability of the finalised 14 item QUAD. The Cronbach’s alpha value for the finalised 14 item QUAD scale was 0.86 indicating good internal consistency. Test-retest reliability remained substantial with a Lin’s concordance of ρ_c_ = 0.81. There was a significant moderate positive correlation between the Stigma Scale and the 14 item version of QUAD (r = 0.39, p < 0.001) and between the ISMI scale and the QUAD (r = 0.45, p < 0.001). The correlation coefficient between the ISMI and the QUAD was within the required threshold of 0.40 to 0.70 [27] and the coefficient between the Stigma Scale and the QUAD was marginal and considered sufficiently near the threshold to indicate adequate convergent validity. In terms of precision, all items satisfied the pre-specified criterion of > 0.30 indicating acceptable fit with the total score of the 14 item scale. The acceptability of the scale remained good with no violation of the MEF and AEF criteria. The Flesch Reading Ease score was 69.1 indicating good acceptability for general adult populations and the Flesch-Kincaid Grade level was 8.2 indicating that it can be understood by 13 to 14 year olds.

### Stage four: cross-replication in an independent sample

The psychometric properties of the finalised QUAD were further established using data from the MIRIAD study. The Cronbach’s alpha value for the 14 item scale used in the MIRIAD study was 0.88 indicating good internal consistency. Convergent validity was assessed using the ISMI and the DISC-12 scale. There was a significant moderate positive correlation between the QUAD and the ISMI scale (r = 0.46, p < 0.001) and between the QUAD and the DISC scale (r = 0.45, p < 0.001). Both correlation coefficients were within the required threshold of 0.40 to 0.70 indicating adequate convergent validity [27]. All items satisfied the pre-specified criterion of > 0.30 for precision, indicating acceptable fit of the 14 items. There were also no violations of the MEF and AEF criteria.

We also conducted an exploratory factor analysis using the MIRIAD study data (180 observations) to examine the factor structure of the 14 item scale. A two factor structure was yielded following inspection of the scree plot with eigen values of 5.49 and 1.4 respectively. Factor 1 explained 35.08% of the variance and contained eight items, all with factor loadings greater than 0.3 (Table 4). These items appeared to refer to institutions/services. Item 2 (people in my neighbourhood) loaded onto this factor suggesting that the stigma or discrimination anticipated within one’s community may be similar to that of more organised institutions. Factor 2 explained 6.1% of the variance and contained six items which appeared to refer to interpersonal/professional relationships. All except item 6 (family) had factor loadings greater than 0.3 but this was considered marginal (0.29) to support the criterion. This item also loaded onto factor 1 which may indicate that the family can be seen as a form of institution. Although this item did not clearly load onto one factor it was retained as it is considered to be an important life domain in which individuals anticipate and experience mental health related discrimination [10,38]. There was a moderate correlation (r = 0.56) between the two factors and the two subscales showed good internal consistency with alpha values of 0.84 and 0.73 respectively.

**Table 4:** Factor loadings from the exploratory factor analysis of the 14 item QUAD (MIRIAD sample)

**QUAD items**	**FACTOR 1 (Institutions/Services)**	**FACTOR 2 (Interpersonal/Professional Relationships)**
11. If religious officials or the community (e.g. at church, mosque or temple) know I have a mental health problem they will treat me unfairly	0.79	
9. If transport drivers and officials (e.g. bus driver, ticket inspector, taxi driver) know about my mental health problem they will treat me unfairly	0.73	
10. If benefit officials know I have a mental health problem they will treat me unfairly	0.69	
13. If physical health staff (e.g. GP, nurse, dentist) know I have a mental health problem they will treat me unfairly	0.55	
12. If the police know I have a mental health problem they will treat me unfairly	0.47	
4. If housing officials or landlords know I have a mental health problem they will treat me unfairly	0.47	
2. If people in my neighbourhood know I have a mental health problem they will treat me unfairly	0.45	
5. If teachers, lecturers or tutors know I have a mental health problem they will treat me unfairly	0.41	
7. If employers know I have a mental health problem they will treat me unfairly		0.77
8. If work colleagues know I have a mental health problem they will treat me unfairly		0.72
3. If a person I want to date or have an intimate relationships with knows I have a mental health problem they will treat me unfairly		0.59
1. If friends know about my mental health problem they will treat me unfairly		0.48
14. If children and teenagers in the community know about my mental health problem they will treat me unfairly		0.43
6. If my family knows about my mental health problem they will treat me unfairly	0.28	0.29

Note: EFA using data from the MIRIAD study. Loadings < 0.3 are suppressed for interpretation. Factor loadings for item 6 were < 0.3; both loadings are presented.

Replication of the factor analysis using the online survey sample in stage two (91 observations) also yielded a two factor structure with eigen values of 5.14 and 1.75 respectively. Factor 1 explained 32.64% of the variance and contained eight items with factor loadings greater than 0.3 (Table 5). Unlike the previous factor structure, item 7 (employers) loaded onto the first factor whilst item 2 (people in my neighbourhood) loaded onto the second factor. However, the items on factor 1 may still be considered to refer to institutions/services as employers may also be viewed in this context. Factor 2 explained 8.7% of the variance and these six items may be seen as referring to interpersonal/professional relationships as 'people in my neighbourhood’ may also be viewed within this context. There was a moderate correlation (r = 0.54) between the two factors and the sub-scales showed good internal consistency with alpha values of 0.82 and 0.76 respectively.

**Table 5:** Factor loadings from the exploratory factor analysis of the 14 item QUAD (Initial sample in Stage 2)

**QUAD items**	**FACTOR 1 (Institutions/Services)**	**FACTOR 2 (Interpersonal/Professional Relationships)**
10. If benefit officials know I have a mental health problem they will treat me unfairly	0.75	
13. If physical health staff (e.g. GP, nurse, dentist) know I have a mental health problem they will treat me unfairly	0.69	
5. If teachers, lecturers or tutors know I have a mental health problem they will treat me unfairly	0.59	
11. If religious officials or the community (e.g. at church, mosque or temple) know I have a mental health problem they will treat me unfairly	0.58	
4. If housing officials or landlords know I have a mental health problem they will treat me unfairly	0.52	
9. If transport drivers and officials (e.g. bus driver, ticket inspector, taxi driver) know about my mental health problem they will treat me unfairly	0.52	
12. If the police know I have a mental health problem they will treat me unfairly	0.49	
7. If employers know I have a mental health problem they will treat me unfairly	0.41	
3. If a person I want to date or have an intimate relationships with knows I have a mental health problem they will treat me unfairly		0.86
2. If people in my neighbourhood know I have a mental health problem they will treat me unfairly		0.81
14. If children and teenagers in the community know about my mental health problem they will treat me unfairly		0.66
8. If work colleagues know I have a mental health problem they will treat me unfairly		0.49
1. If friends know about my mental health problem they will treat me unfairly		0.45
6. If my family knows about my mental health problem they will treat me unfairly		0.29

Note: EFA using data from the online survey described in Stage 2. Loadings < 0.3 are suppressed for interpretation.

## Discussion

This study presents findings on the development and finalisation of a measure of the extent to which people with mental health problems anticipate personally experiencing mental health-related discrimination across various life domains and its psychometric properties. Prior to the development of the QUAD, there was no existing measure addressing this vital concept [39], that may have serious detrimental effects on people with mental health problems. Consequently the QUAD is an important addition to the portfolio of measures addressing aspects of stigma and discrimination. The finalised 14 item QUAD was found to have good internal consistency, test-retest reliability, adequate convergent validity and good acceptability for general adult populations. Cross-replication in an independent sample provided further evidence of its reliability, acceptability and validity. The scale can be viewed as consisting of two sub-scales summarised as anticipated discrimination from institutions/services and anticipated discrimination in interpersonal/professional relationships. The most highly reported areas of life in which participants anticipated discrimination were related to 'children and teenagers in my community’, and 'employers and work colleagues’. Abuse by neighbourhood youth has been noted in a recent overview of targeted violence and harassment against people with mental health problems [20]. The frequent reporting of anticipated discrimination from employers and work colleagues echoes the finding that employment is an area in which individuals have often stopped themselves from engaging in due to the anticipation of unfair treatment [6,7], sometimes because of having experienced this [40]. This may lead to decisional conflict at work, concealment of mental health problems and the non-receipt of reasonable adjustments [41-43].

### Strengths and limitations

A major strength of this study is the multi-stage iterative process used to develop the measure. Unlike other measures, the newly developed measure covers a wider range of life contexts in which discrimination may be personally anticipated as opposed to more general contexts. It is differentiated from perceived discrimination scales in that it assesses the extent to which people anticipate discrimination directed at themselves rather than at people with mental health problems in general. Furthermore, the QUAD is differentiated from the anticipated discrimination section of the DISC in that the former assesses anticipation itself whereas the latter focuses on responses to anticipation. A further strength is that the scale is both short and simple to administer amongst its intended population as it was designed as a self-completion measure which received a positive overall rating by respondents. In addition, the assessment of psychometric properties was comprehensive in that it included concepts such as precision and maximum endorsement frequencies. The psychometric properties were also further established in an independent sample in order to provide further information about the psychometric properties of the scale.

A limitation of the study is that the sample for the online survey was recruited through mental health-related organisations, one being an anti-stigma campaign (Time to Change) [44] and so may be atypical. These individuals may be more likely to anticipate discrimination and may be potentially engaged in efforts to reduce discrimination. The online sample is also limited to those who have internet access and the use of a computer. Males, individuals from Black and minority ethnic groups and those with a diagnosis of schizophrenia/schizoaffective disorders were under-represented in this sample. However, the MIRIAD sample overcomes these limitations as participants were mental health service users engaging with community mental health teams. The sample includes people with psychosis and is more varied in terms of ethnicity and gender. The MIRIAD sample was also large enough to conduct an exploratory factor analysis in order to provide further information about the construct validity of the QUAD items. In addition, although the sample in the online survey was small in number and atypical, the exploratory factor analysis using this sample provides a preliminary replication of the factor structure found in the independent sample.

### Application

The QUAD may be used to assess an individual’s level of anticipated discrimination which is likely to be an important addition in both research and clinical contexts. We recommend general use of the total score as the overall scale has demonstrated good reliability and validity. Where the research question deems it appropriate, subscales scores from the MIRIAD sample may also be used. The QUAD can also be used to identify key life domains in which people personally anticipate mental health related discrimination which may highlight areas of focus when developing anti-stigma initiatives. It may be used as a complementary scale to measures of experienced discrimination such as the Discrimination and Stigma Scale (DISC-12) [10] as well as in conjunction with other measures assessing the behavioural responses to both anticipated and experienced discrimination such as the Stigma Scale [14] and the Perceived Devaluation and Discrimination Scale [12]. The 14 item QUAD is available for use (see Additional file 1) together with its manual and conditions of use from http://www.sapphire.iop.kcl.ac.uk[17]. Queries should be addressed to the corresponding and/or last author.

### Implications for future research

Although the psychometric properties of the QUAD have been established in an independent sample, additional research would be beneficial in order to provide further evidence of the validity of the scale. This includes further replication of the exploratory factor analysis and a confirmatory factor analysis in order to establish whether the identified two factor structure from the MIRIAD sample can be replicated. Further research is also required to establish how the QUAD relates to measures of experienced discrimination and internalised stigma in order to further investigate the association between the three concepts. Such research questions are being addressed by our research group using data from the MIRIAD study.

## Conclusions

The QUAD is a newly developed measure which can be used to assess levels of anticipated discrimination amongst people with mental health problems and this study provides preliminary evidence for its reliability, validity and acceptability. The scale expanded upon previous versions of the DISC [6,18] and upon further work by our group examining areas of life where discrimination may be personally experienced, followed by focus groups and cognitive - debriefing interviews. The items were finalised following an online survey of 117 mental health service users, resulting in a comprehensive measure of anticipated discrimination which covers a broad range of contexts in which mental health discrimination may be anticipated. The QUAD can be used to identify the key life domains in which people personally anticipate discrimination and may be useful in evaluating interventions aimed at reducing the stigma of mental illness.

## Competing interests

The authors declare that they have no competing interests.

## Authors’ contributions

EB designed and led the study, drafted the versions of the questionnaire, collected the data and contributed to the data interpretation. JG conducted the main analysis, interpreted the data and drafted the manuscript. SC supervised the study, contributed to study design, questionnaire development, and data interpretation. CH and GT contributed to study design, questionnaire development, and data interpretation. All authors critically revised the draft manuscript, and read and approved the final manuscript.

## Authors’ information

Jheanell Gabbidon and Elaine Brohan are joint first authors.

## Pre-publication history

The pre-publication history for this paper can be accessed here:

http://www.biomedcentral.com/1471-244X/13/297/prepub

## Supplementary Material

Additional file 1 Questionnaire on anticipated discrimination. Click here for file
