# Supplementary material for: The development and validation of the Questionnaire on Anticipated Discrimination (QUAD)
Source: BMC Psychiatry. 2013 Nov 7;13:297. doi: 10.1186/1471-244X-13-297 (PMC4226195; doi:10.1186/1471-244X-13-297)
Supplement: Additional file 1 — Questionnaire on anticipated discrimination. [file 1471-244X-13-297-S1.pdf]

Questionnaire on Anticipated Discrimination **QUAD**

Instructions: Below you will find a list of sentences. For each one, please indicate the answer that best suits you by circling the number in the appropriate box. Don't spend too much time thinking about the answer, as it is your first impression that is important.

	Statement	strongly disagree	disagree	agree	strongly agree
1	If friends know about my mental health problem, they will treat me unfairly.	0	1	2	3
2	If people in my neighbourhood know I have a mental health problem, they will treat me unfairly.	0	1	2	3
3	If a person I want to date or have an intimate relationship with knows I have a mental health problem, they will treat me unfairly.	0	1	2	3
4	If housing officials or landlords know I have a mental health problem, they will treat me unfairly.	0	1	2	3
5	If teachers, lecturers or tutors know I have a mental health problem, they will treat me unfairly.	0	1	2	3
6	If my family knows about my mental health problem, they will treat me unfairly.	0	1	2	3
7	If employers know I have a mental health problem, they will treat me unfairly.	0	1	2	
8	If work colleagues know I have a mental health problem, they will treat me unfairly.	0	1	2	3
9	If transport drivers and officials (e.g. bus driver, ticket inspector, taxi driver) know about my mental health problem, they will treat me unfairly.	0	1	2	3
10	If benefit officials know I have a mental health problem, they will treat me unfairly.	0	1	2	3
11	If religious officials or the community (e.g. at church, mosque or temple) know I have a mental health problem, they will treat me unfairly.	0	1	2	3
12	If the police know I have a mental health problem, they will treat me unfairly.	0	1	2	3
13	If physical health staff (e.g. GP, nurse, dentist) know I have a mental health problem, they will treat me unfairly.	0	1	2	3
14	If children and teenagers in my community know I have a mental health problem, they will treat me unfairly.	0	1	2	3

Questionnaire on Anticipated Discrimination QUAD © 2011. Health Service and Population Research Department, Institute of Psychiatry, King's College London.
We would like to thank Elaine Brohan for her major contribution to the development of this scale.
Contact: Professor Graham Thornicroft, graham.thornicroft@kcl.ac.uk
